# Supplementary material for: Molecular Subtyping of Invasive Breast Cancer Using a PAM50-Based Multigene Expression Test-Comparison with Molecular-Like Subtyping by Tumor Grade/Immunohistochemistry and Influence on Oncologist’s Decision on Systemic Therapy in a Real-World Setting
Source: Int J Mol Sci. 2022 Aug 5;23(15):8716. doi: 10.3390/ijms23158716 (PMC9368794; doi:10.3390/ijms23158716)
Supplement: Supplementary file 1 [file ijms-23-08716-s001.zip › ijms-1824104-supplementary.pdf]

Supplementary Material

Tissue processing and routine diagnostics

For diagnosis of IBC, tumor tissue was formalin fixed and embedded (FFPE) according to the local standards. Hematoxylin and eosin (H&E) stained slides of the tumor area were evaluated by pathologists experienced in breast cancer pathology regarding histological subtype, tumor grade (according to Elston & Ellis [1]), associated in situ component, TNM classification, angioinvasion, and resection margins according to aforementioned guidelines.

Immunohistochemistry and HER2 chromogenic in situ hybridization (CISH)

"Decentral" local IHC staining of ER, PR, and Ki-67 protein expression, as well as assessment of HER2 status, were performed in decentral laboratories according to their local standards, (inter-)national guidelines and manufacturer's recommendations. For the "central" assessment in center C1, staining was performed on 1 μm thick sections of formalin fixed paraffin embedded (FFPE) tumor blocks on a Ventana BenchMark Ultra automated instrument (Ventana Medical Systems, Inc., Oro Valley, AZ, USA). The following antibodies were used for evaluation: for ER the monoclonal Rabbit Anti-Human ER α, Clone EP1 (dilution 1:40), for PR the monoclonal Mouse Anti-Human Progesterone Receptor, clone PgR 636 (dilution 1:50), for HER2 the polyclonal Rabbit Anti-Human c-erbB-2 Oncoprotein (dilution 1:1000) and for Ki-67 the monoclonal Mouse Anti-Human Ki-67 Antigen, Clone MIB-1 (dilution 1:100) (all from Dako, Agilent Technologies, Santa Clara, CA, USA). Heat-induced epitope retrieval (HIER) was performed using CC1 at 95^{0}C for 36 min followed by antibody incubation at 37^{0}C for 32 min for the ER, PR, and HER2 antibodies, whereas for the Ki-67 antibody HIER was performed using CC1 at 95^{0}C for 52 min followed by antibody incubation at 37^{0}C for 32 min. Binding of the antibodies to the antigen was visualized using the ultraView Universal DAB Detection Kit and, subsequently, sections were counterstained with Haematoxylin and Bluing Reagent (Ventana). IBC was defined as ER and PR positive if ≥ 1% of the tumor nuclei were stained positively [2,3]. Ki-67 was measured as percentage of positively stained tumor nuclei according to in-house established methods

(C1: hot spot method). HER2 assessment was done according to Hercep Test criteria and scored as 0, 1+, 2+, or 3+. If HER2 IHC score was 2+, reflex testing [*HER2* chromogenic in situ hybridization (CISH)] was ordered. Each laboratory performed *HER2* CISH according to their local standards. In C1, the Zyto*Dot*® 2C SPEC *ERBB2/CEN 17* Probe (ZytoVision GmbH, Bremerhaven, Germany) was used after pepsin pretreatment (37^{0}C, 7 min) and followed by hybridization overnight at 37^{0}C. A *HER2/CEN17* ratio ≥ 2.0 or an average HER2 copy number per nuclei ≥ 6 defined HER2 amplification, and thus positivity [4].

Statistical analysis

Out of the initial 142 IBC cases, 23 cases were removed from the dataset: n = 2 recurrent tumors, n = 3 triple negative surrogate subtype IBCs, and n = 18 IBCs not reporting any surrogate subtyping (neither locally nor centrally assessed). Thus, a total of 119 tumor cases were used for statistical analyses. The Cohen's Kappa coefficient (κ) was used as statistic to measure pairwise agreement between Prosigna® subtypes and IHC / IHC+G subtypes (i.e. Prosigna® vs. local assessments, Prosigna® vs. C1 assessments and Prosigna® vs. C6 assessments) relying on the function *kappa2* of the R package *irr* v.0.84.1 [5]. To this aim, both Prosigna® subtyping and molecular-like subtypes were re-mapped into a 4-tiered coding system (Table S1). κ values <0.0, 0.0-0.2, 0.21-0.4, 0.41-0.6, 0.61-0.8 and 0.81-1 indicated "poor", "slight", "fair", "moderate", "substantial" and "almost perfect" strength of agreement [6]. The Kruskal-Wallis statistical test was used to evaluate the differences in Ki-67 expression between the three assessment centers (i.e. local, C1 and C6). Post-hoc pairwise comparisons between local and central (C1 and C6) Ki-67 expression values were performed relying on the non-parametric Wilcoxon test for independent samples adjusting p-values via the Benjamini-Hochberg (B-H) method. Pearson's chi-squared (χ^2) test was used to analyze the association between categorical variables. For those cases where the assumptions underlying χ^2-test, i.e. more than five expected counts in at least 80% of the cells and no cell with less than one expected count [7]), were not met, two-sided Fisher's exact test was used. P-values (or, where applicable, adjusted p-values) < 0.05 were considered statistically significant.

Supplementary Tables

Table S1. 4-tiered coding system utilized to measure pairwise agreement between the different subtyping methods. Luminal A Prosigna® molecular subtype, Luminal A-like IHC molecular-like subtype and Luminal A-like IHC+G molecular-like subtype were encoded with 1. Luminal B Prosigna® molecular subtype, Luminal B-like (HER2-) IHC molecular-like subtype and Luminal B-like HER2- IHC+G molecular-like subtype were encoded with 2. HER2 enriched Prosigna® molecular subtype, Luminal HER2+ IHC molecular-like subtype and Luminal B-like HER2+ IHC+G molecular-like subtype were encoded with 3. Basal like Prosigna® molecular subtype was encoded with 4.

**Subtyping**	**Original subtypes**	**New coding**
Prosigna®	Luminal A	1
IHC	Luminal A-like	
IHC+G	Luminal A-like	
Prosigna®	Luminal B	2
IHC	Luminal B-like	
IHC+G	Luminal B-like HER2-	
Prosigna®	HER2 enriched	3
IHC	Luminal HER2+	
IHC+G	Luminal B-like HER2+	
Prosigna®	Basal like	4

IHC: immunohistochemistry; IHC+G = immunohistochemistry + tumor grade.

Table S2. Summary of age at diagnosis, tumor size, Ki-67 expression and tumor grade. For continuous variables the range and the median value are provided, whereas for discrete variables, the proportion with respect to the total number of available cases is reported.

Variable	Range	Median	Center	Proportion (%)
Age at diagnosis	39-78 years	55 years	//	//
Tumor size	0.7-15.4 cm	1.8 cm	//	//
Ki-67	1-80 %	20	Locally assessed	//
	1-60 %	20	C1 assessed	//
	1-60 %	12.5	C6 assessed	//
Tumor grade	//	//	Locally assessed	G1: 4/118 (3.4%)<br>G2: 82/118 (69.5%)<br>G3: 32/118 (27.1%)
			C1 assessed	G1: 2/110 (1.8%)<br>G2: 70/110 (63.6%)<br>G3: 38/110 (34.5%)
			C6 assessed	G1: 21/109 (19.3%)<br>G2: 81/109 (74.3%)<br>G3: 7/109 (6.4%)

Ki-67: proliferation marker.

Supplementary Figure Legends

Figure S1. Distribution of tumor grade across the different centers. Within each center barplots indicate the number of cases detected for each tumor grade.

Figure S2. Distribution of Ki-67 expression across the different centers. Adjusted p-values are based on post-hoc pairwise two-sided Wilcoxon test for independent samples. ****: adjusted p-value < 0.0001; ns: non-significant (adjusted p-value > 0.05).

Figure S3. Relationship between Ki-67 expressions reported by centers C1 and C6. A) IHC+G Luminal A-like cases detected in center C6 and coloured according to the respective assessment in C1; B) IHC+G Luminal B-like HER2- cases detected in center C1 and coloured according to the respective assessment in C6. IHC+G = immunohistochemistry + tumor grade; LumA = Luminal A; LumB = Luminal B.

Figure S4. Distribution of C1 tumor grade assessments across C6 Luminal A-like IHC+G subtypes graded by C6 as A) G1 or B) G2. IHC+G = immunohistochemistry + tumor grade; LumA = Luminal A; LumB = Luminal B.

Supplementary Figures

Figure S1. Distribution of tumor grade across the different centers. Within each center barplots indicate the number of cases detected for each tumor grade.

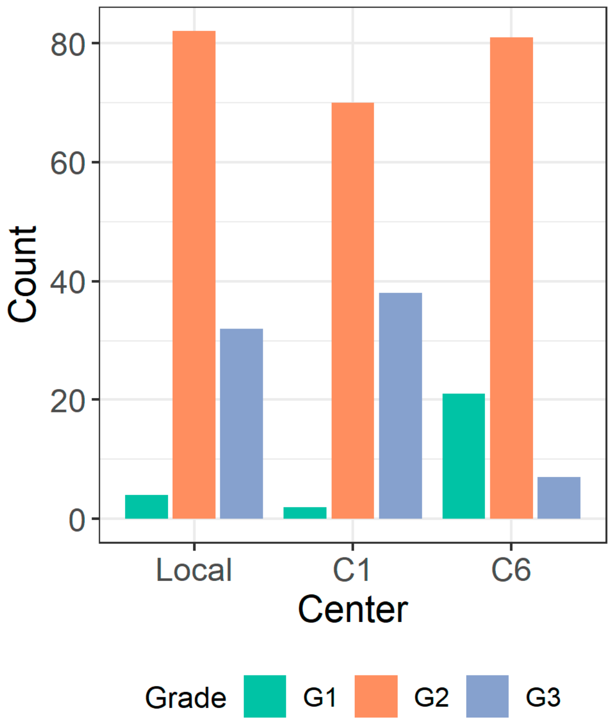

Figure S2. Distribution of Ki-67 expression across the different centers. Adjusted p-values are based on post-hoc pairwise two-sided Wilcoxon test for independent samples. ****: adjusted p-value < 0.0001; ns: non-significant (adjusted p-value > 0.05).

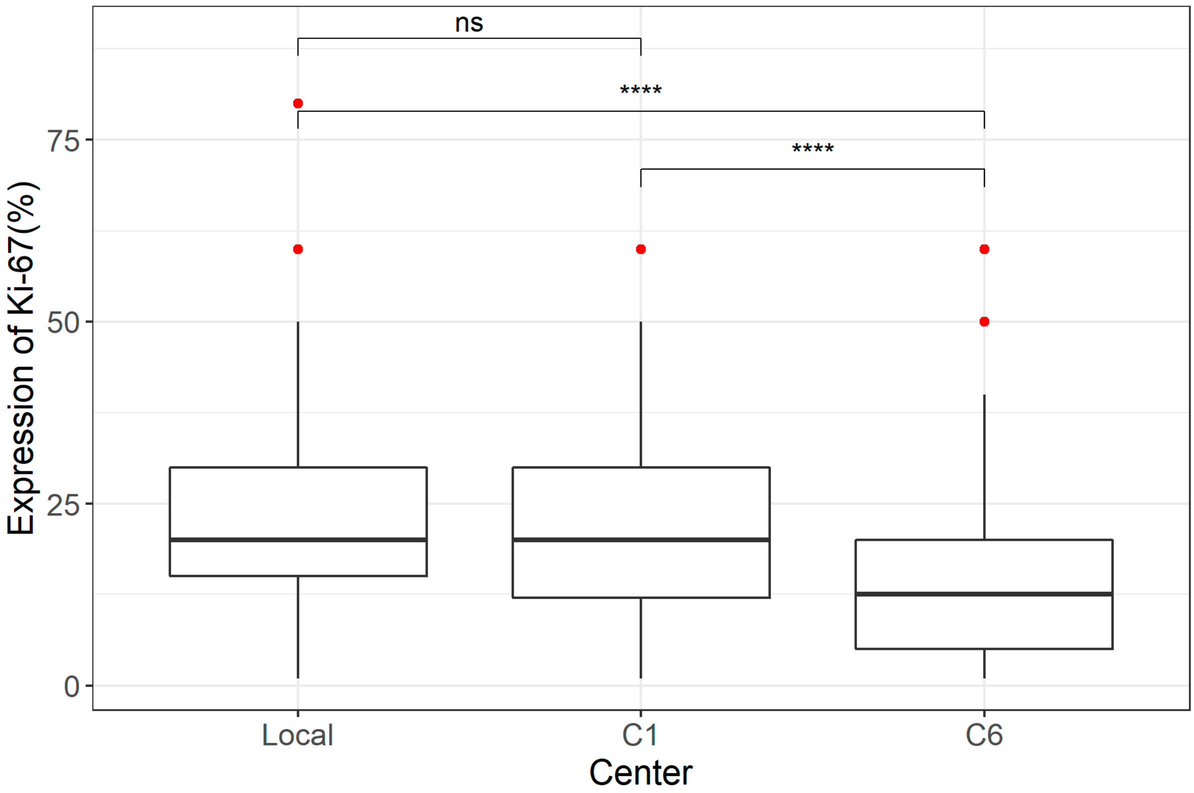

Figure S3. Relationship between Ki-67 expressions reported by centers C1 and C6. A) IHC+G Luminal A-like cases detected in center C6 and coloured according to the respective assessment in C1; B) IHC+G Luminal B-like HER2- cases detected in center C1 and coloured according to the respective assessment in C6. IHC+G = immunohistochemistry + tumor grade; LumA = Luminal A; LumB = Luminal B.

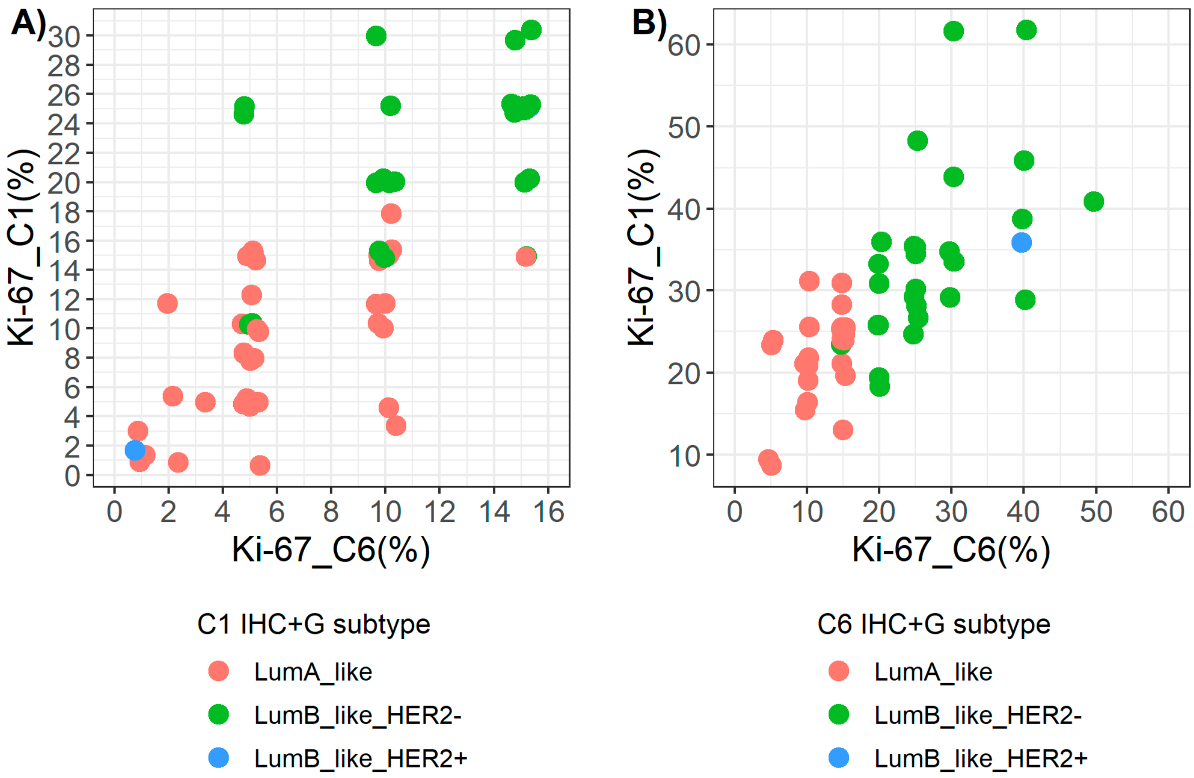

Figure S4. Distribution of C1 tumor grade assessments across C6 Luminal A-like IHC+G subtypes graded by C6 as A) G1 or B) G2. IHC+G = immunohistochemistry + tumor grade; LumA = Luminal A; LumB = Luminal B.

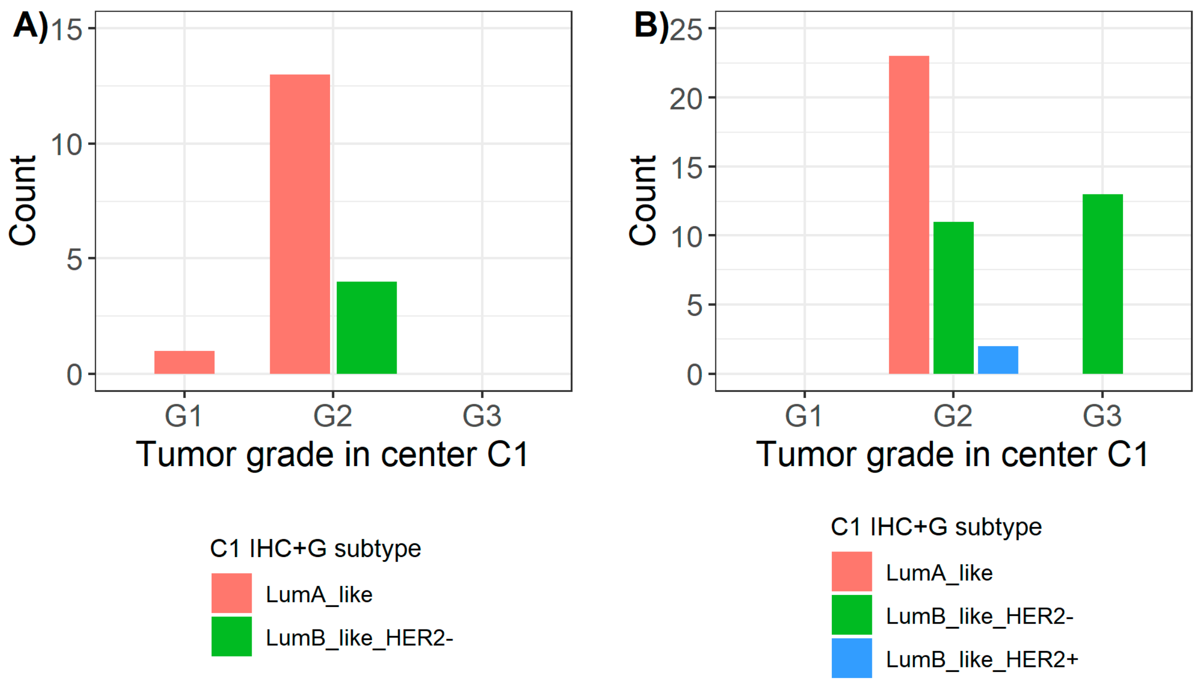

References

1. Elston, C.W.; Ellis, I.O. Pathological prognostic factors in breast cancer. I. The value of histological grade in breast cancer: experience from a large study with long-term follow-up. *Histopathology* **1991**, *19*, 403–410.
2. Allison, K.H.; Hammond, M.E.H.; Dowsett, M.; McKernin, S.E.; Carey, L.A.; Fitzgibbons, P.L.; Hayes, D.F.; Lakhani, S.R.; Chavez-MacGregor, M.; Perlmutter, J.; et al. Estrogen and Progesterone Receptor Testing in Breast Cancer: ASCO/CAP Guideline Update. *J. Clin. Oncol.* 2020, 38, 1346–1366. https://doi.org/10.1200/jco.19.02309.
3. Hammond, M.E.; Hayes, D.F.; Dowsett, M.; Allred, D.C.; Hagerty, K.L.; Badve, S.; Fitzgibbons, P.L.; Francis, G.; Goldstein, N.S.; Hayes, M.; et al. American Society of Clinical Oncology/College Of American Pathologists guideline recommendations for immunohistochemical testing of estrogen and progesterone receptors in breast cancer. *J. Clin. Oncol.* **2010**, *28*, 2784–2795. https://doi.org/10.1200/jco.2009.25.6529.
4. Wolff, A.C.; Hammond, M.E.; Hicks, D.G.; Dowsett, M.; McShane, L.M.; Allison, K.H.; Allred, D.C.; Bartlett, J.M.; Bilous, M.; Fitzgibbons, P.; et al. Recommendations for human epidermal growth factor receptor 2 testing in breast cancer: American Society of Clinical Oncology/College of American Pathologists clinical practice guideline update. *J. Clin. Oncol.* **2013**, *31*, 3997–4013. https://doi.org/10.1200/jco.2013.50.9984.
5. Gamer, M.; Lemon, J.; Fellows, I.; Singh, P. irr: Various Coefficients of Interrater Reliability and Agreement. 2019. Available online: https://CRAN.R-project.org/package=irr (accessed on 26 January 2019).
6. Landis, J.R.; Koch, G.G. The measurement of observer agreement for categorical data. *Biometrics* **1977**, *33*, 159–174.
7. Bewick, V.; Cheek, L.; Ball, J. Statistics review 8: Qualitative data–tests of association. *Crit. Care* 2003, 8, 1–8.
